# Rapid Automatized Naming as a Universal Marker of Developmental Dyslexia in Italian Monolingual and Minority-Language Children

**DOI:** 10.3389/fpsyg.2022.783775

**Published:** 2022-04-07

**Authors:** Desiré Carioti, Natale Stucchi, Carlo Toneatto, Marta Franca Masia, Martina Broccoli, Sara Carbonari, Simona Travellini, Milena Del Monte, Roberta Riccioni, Antonella Marcelli, Mirta Vernice, Maria Teresa Guasti, Manuela Berlingeri

**Affiliations:** ^1^Department of Humanities, University of Urbino Carlo Bo, Urbino, Italy; ^2^Department of Psychology, University of Milano-Bicocca, Milan, Italy; ^3^Center of Developmental Neuropsychology, ASUR Marche, Pesaro, Italy; ^4^NeuroMi, Milan Center for Neuroscience, Milan, Italy

**Keywords:** RAN, minority language, heritage language, reading skills, developmental dyslexia (DD)

## Abstract

Rapid Automatized Naming (RAN) is considered a universal marker of developmental dyslexia (DD) and could also be helpful to identify a reading deficit in minority-language children (MLC), in which it may be hard to disentangle whether the reading difficulties are due to a learning disorder or a lower proficiency in the language of instruction. We tested reading and rapid naming skills in monolingual Good Readers (mGR), monolingual Poor Readers (mPR), and MLC, by using our new version of RAN, the RAN-Shapes, in 127 primary school students (from 3rd to 5th grade). In line with previous research, MLC showed, on average, lower reading performances as compared to mGR. However, the two groups performed similarly to the RAN-Shapes task. On the contrary, the mPR group underperformed both in the reading and the RAN tasks. Our findings suggest that reading difficulties and RAN performance can be dissociated in MLC; consequently, the performance at the RAN-Shapes may contribute to the identification of children at risk of a reading disorder without introducing any linguistic bias, when testing MLC.

## Introduction

### Developmental Dyslexia or Socio-Linguistic Disadvantage? The Case of Minority-Language Children in Italy

In the last 20 years, the constant migration flows from different countries significantly reshaped the Italian school’s environment, converting it into a heterogeneous mixture of cultures (Cesareo, 2014). Based on the annual data published by the Italian Ministry of University Education and Research^1^, 842,000 immigrants of first and second-generation were attending Italian public schools of every grade in the year 2017/2018, accounting for 9.7% of the total student population, with an annual increase of the 1.9%. In particular, 11.2% of primary students in 2017/2018 is not of Italian nationality and, in general, 63.1% of these students is born in Italy from foreign parents, mainly native of Romania (18.8%), Albany (13.6%), Morocco (12.3%), and China (6.3%). This suggests that many non-Italian students are second-generation immigrant children with an early bilingual experience. These children generally use the Italian language at school or during extra-familial social activities and their parents’ language at home, and, for this reason, we referred to them as minority-language children (MLC). These children could also be considered as “heritage language” children (Valdés, 2005; Polinsky and Kagan, 2007), as they have learned the minority language outside their parents’ country, while not all of them are proficient in the minority language. However, to properly define a heritage language speaker, it is necessary to be aware of his/her language proficiency in L1; we might not be sure that our multilingual participants can be univocally defined as heritage language children. For this reason, we defined the group based on speakers’ use and exposure to a minority language at home.

Whether the minority language exposure and, in general, a bilingual background might exert an impact on their learning and academic achievements is still under debate (Cobo-Lewis et al., 2002; Kovelman et al., 2015, 2008; Kremin et al., 2019).

It is worthy to note that in 2017/2018, approximately 2% of primary school students reported learning disorders, and it cannot be excluded that some of these students were first or second-generation immigrant children. To date, the diagnostic criteria for learning disorders in minority-language students, who are usually bilinguals, are not currently available in Italy, and often, reading skills are tested with tests standardized on the Italian-native population (Gasperini, 2013; Celentin and Daloiso, 2017). Nevertheless, as widely studied in research (Bialystok, 2006, 2007 for reviews), bilingual students reported some specific cognitive features in terms of both advantages and disadvantages, leading to the need of evaluating these students with *ad hoc* instruments.

Several studies on bilingual speakers have reported limitations on a range of different cognitive and linguistic skills (i.e., smaller vocabularies and weaker access to lexical items) in the face of enhanced executive control (Bialystok, 2001; Abu-Rabia and Siegel, 2002; Bialystok et al., 2004). In particular, Barac and Bialystok (2012) showed that 6-year-old bilingual students may have an advantage in executive functions as compared to monolingual students, while language proficiency is moderated by the type of spoken languages, and, in particular, by the degree of overlap between L1 and L2, and the cultural background of speakers.

Similar findings were reported for bilinguals belonging to minorities (Engel de Abreu, 2011; Engel de Abreu and Gathercole, 2012; Bosma et al., 2017; Hopp et al., 2019). Interestingly, Engel de Abreu et al. (2014) showed that the environmental conditions could negatively influence immigrant students’ language skills, especially concerning vocabulary.

Kovelman et al. (2008) suggested that the influence of the minority language on the acquisition and the expertise of the majority would be moderated by the number of years spent in the country of origin. If this were the case, then, second-generation immigrants would be more advantaged than first-generation immigrants in achieving a proficiency level similar to that of the natives (Portes and Rumbaut, 2005).

Nevertheless, the results about the comparison of natives and second-generation immigrants are not consistent: the academic achievements of this last group do not always match those of natives (Heath et al., 2008; Ortiz and Telles, 2017), and some authors talk of an “immigrant paradox” referring to “the phenomenon that the achievement gaps with monolingual peers widen for later generations” (Prevoo et al., 2016, p. 241). Despite all this evidence about linguistic aspects, less is known concerning the relationship between language proficiency and MLC learning outcomes.

Focusing on the Italian situation, the research conducted by Barban and White (2011) on immigrant students revealed a sort of linear decreasing trend: first and second-generation immigrants have poorer outcomes than natives in the middle school final exam, but the second-generation shows a higher level of performance when compared to first-generation immigrants. In general, this pattern was moderated by the time gap between their arrival in Italy and their examination day.

However, when Azzolini et al. (2012) explored reading and math skills of first- and second-generation immigrants and children with at least one foreign parent in Italy and Spain, using data of the PISA 2009^2^, they observed that both first- and second-generation immigrants in Spain underperformed controls for literacy. On the other hand, in Italy, the gap between performances of natives and first-generation immigrants was higher than the one between natives and second-generation immigrants, but the discrepancy between first- and second-generation immigrants became approximately zero when controlling for socioeconomic status and hours spent speaking the minority language (Azzolini et al., 2012). These results are in line with Murineddu et al. (2006), who had already reported difficulties for foreign children compared to Italian native speakers in literacy, but not in math abilities. More recently, Bellocchi et al. (2016) pointed out that the vocabulary size of L2 moderates bilingual readers’ performances in Italian.

From the clinical point of view, the MLC’s difficulties in acquiring and managing literacy abilities often make this population close to profiles of learning disabilities, but whether this comparison is justified is still to be proven. Scortichini et al. (2012) pointed out that the cognitive and learning profile of bilingual students labeled as “Learning disabled” was significantly different from the one of Italian dyslexic readers.

Reading difficulties observed in bilingual children were related to a weaker lexical and orthographic recognition, probably due to a more impoverished Italian vocabulary. However, the authors noted that given the differential profiles that emerged, pooling together all children with reading difficulties regardless of their linguistic context, would decrease the chance to take appropriate and specific actions for bilingual students with learning difficulties and children with developmental dyslexia (DD) (Scortichini et al., 2012). Considering all this evidence, the question that emerges is whether MLC’s reading difficulties in primary school can be ascribed to a learning disorder and not to a generic linguistic weakness. From this perspective, it is crucial to discern between a learning disorder and language disadvantage due to a limited exposure to the Italian language to establish clear diagnostic criteria, improve clinical practices, and enhance school outcomes of MLC.

### The Rapid Automatized Naming-Reading Relationship

Students speaking more than one language, who struggle at school, might be misclassified as having a learning disorder even in the absence of a real neurodevelopmental deficit. As suggested above, this can be because, on average, multilingual students may obtain lower performance in reading tasks as a consequence of language disadvantages rather than DD. The purpose of the current paper is to empirically investigate whether a well-known task, the RAN, might also be a candidate clinical marker of a learning disorder in the bilingual population (specifically in MLC).

The RAN has a long history in research toward acquired and congenital reading disabilities, such as alexia and DD. The task, developed by Denckla and Rudel (1976) in three formats (objects, digits, and letters), is mainly referred to as the ability to rapidly recall and name a range of limited stimuli in an array. From its first creation, RAN has been repeatedly adopted in the context of reading and DD, and some methodological aspects as (i) the number and type of stimuli, and (ii) the dimension of the grid, were largely tested in these last 50 years (refer to, for example, Georgiou et al., 2013; Georgiou and Parrila, 2020; refer to Norton and Wolf, 2012 for a review). The results suggest that the RAN is one of the most reliable universal predictors of reading difficulties (refer to Kirby et al., 2010; Araújo et al., 2015; Araújo and Faísca, 2019), irrespective of the specific presentation features. In this regard, Wolf and Bowers’ Double Deficit hypothesis (1999) states that both phonological deficits, as well as processes underlying naming speed, might be accounted as separable sources of reading deficits. Consequently, a deficit in at least one of the two domains may compromise their reading performance giving rise to different profiles of DD. It is important to note, however, that the data collected in a shallow orthography language, such as Italian, showed that RAN can be considered as the main cognitive marker of DD, in contrast to phonological awareness (Brizzolara et al., 2006). According to these authors, the DD children without a previous language delay scored in the average range on most phonologic tasks, while a RAN deficit was the most frequent deficit shared by children with dyslexia with and without a previous language delay.

Wolf and Bowers (1999) further suggested that rapid naming is a complex ability involving several cognitive operations in common with reading. These operations include attentional processes, bi-hemispheric visual processes, integration of visual and orthographic features, phonological and lexical retrieval, recall and integration of semantic information, and, lastly, motor planning and consequent articulation of the vocal output (Wolf and Bowers, 1999). In such a multifaceted picture, the strength of the relation between RAN and reading has been repeatedly observed and reported (refer to Denckla and Cutting, 1999; Norton and Wolf, 2012 for reviews), but after more than 20 years, its cognitive nature is still under investigation.

There is a long tradition of attempting to discern the relationship between RAN and reading; some studies (Scarborough, 1998; Cunningham, 2006) found small or no correlation between these two cognitive abilities, while, in line with the previous meta-analytic results of Swanson et al. (2003), the meta-analysis by Araújo et al. (2015) found a moderate-to-strong correlation between RAN and reading measures, that was confirmed in a more recent meta-analytic work of the same group (Araújo and Faísca, 2019).

Nevertheless, a wide range of questions about the specific cognitive features shared by RAN and reading remains debated in the literature.

One of the main issues in the study of the RAN-reading relationship is to disentangle whether the RAN is related to the phonological or visual-orthographic processes underlying reading skills (refer to Araújo et al., 2015; Araújo and Faísca, 2019), while a further matter of debate is focused on whether the RAN predicts fluency or accuracy reading measure. Although there is vast agreement about the fact that the RAN mostly predicts reading fluency, this empirical evidence mostly belongs to studies in transparent orthographies, like Italian, German or Greek compared to English (Landerl and Wimmer, 2000; Di Filippo et al., 2005; Nikolopoulos et al., 2006), and could be, thus, attributed to orthographic consistency more than to a mere link between reading speed and serial naming speed (Georgiou et al., 2008b); a hypothesis that has not been tested by the meta-analytic results of Araújo and Faísca (2019).

Another relevant feature that RAN and reading might share, and that can slow down the performance of dyslexic readers, is the sensitivity to the crowding effect (Moll and Jones, 2013). Crowding is known as the effect for which the detection of an object is even more difficult when it is surrounded by other objects (Bouma, 1970), and several studies support the idea that dyslexic readers tend to be more sensitive to this type of visuo-attentional effect (refer to Gori and Facoetti, 2015 for a review). This is not surprising if we consider that the reading process, as well as the sequential rapid naming, implies foveal and parafoveal processing (Jones et al., 2009). The inter-letters spacing showed to be a relevant variable for inducing crowding in DD readers (Spinelli et al., 2002; Yu et al., 2007; Martelli et al., 2009) and, in general, this effect seems to be related to a failure in selective attention and, thus, inhibition of interference (refer to Bellocchi, 2013; Gori and Facoetti, 2015).

By investigating eye movements during a RAN-letter task in different conditions of inter-letter spacing, Moll and Jones (2013) concluded that dyslexic readers are affected by crowding at the level of both foveal and parafoveal processing. In line with these pieces of evidence, we adopted in this study a new version of RAN, the RAN-Shapes, in which this aspect is considered and further investigated by manipulating shape dimension and perceptual properties of each matrix presented to our participants.

### Rapid Automatized Naming as a Cross-Linguistic Marker of Developmental Dyslexia

As assumed by the Orthographic Depth Hypothesis (Katz and Frost, 1992) and the Grain Size Theory (Ziegler and Goswami, 2005), reading acquisition and development can vary depending on grapheme to phoneme correspondence, and on the complexity of the orthography (Aro and Wimmer, 2003; Seymour et al., 2003; Ziegler and Goswami, 2006; Carioti et al., 2021). Therefore, the reading experience is sensitive to the orthographic depth of each language. In this perspective, to establish whether the RAN predicting power can be influenced by the orthography is crucial to define it as a universal and cross-linguistic predictor of reading.

As summarized by many reviews and meta-analyses (Denckla and Cutting, 1999; Kirby et al., 2010; Norton and Wolf, 2012), the role of different versions of the RAN in predicting reading outcomes and in discerning between non-impaired and impaired readers emerged both for shallow (Di Filippo et al., 2005; Heikkilä et al., 2009; Jones et al., 2009, 2016; Lervåg and Hulme, 2009; Moll and Landerl, 2009; Torppa et al., 2013; Zoccolotti et al., 2013; Tobia and Marzocchi, 2014; Rodríguez et al., 2015) and deep orthographies (Savage et al., 2007; Arnell et al., 2009; Georgiou et al., 2011; Vander Stappen et al., 2020), as well as in non-alphabetic languages (Yan et al., 2013; Pan and Shu, 2014; Georgiou and Parrila, 2020; Gharaibeh et al., 2021).

Further support comes from studies (Vaessen et al., 2010; Ziegler et al., 2010; Landerl et al., 2013; Moll et al., 2014) that have investigated the cross-cultural role of reading predictors, i.e., RAN and Phonological Awareness (PA). These studies concluded that both abilities represent the universal core deficits of dyslexia (Parrila et al., 2020). Confirming this picture, a recent meta-analysis (Carioti et al., 2021) found a systematic difference in non-impaired and dyslexic readers for PA and RAN both in shallow and deep orthographies, in children and adult readers.

Some studies by Georgiou (Georgiou et al., 2008a,^2016^) found that the performance at RAN predicts reading skills across orthographies and alphabetic vs. non-alphabetic systems. Even if the differences in predicting 2nd-graders’ reading outcomes were found in English and Greek students, depending on the use of an alphanumeric or a non-alphanumeric RAN task, the RAN-Digits significantly contributed to reading fluency in both languages (Georgiou et al., 2008c). Also including a non-alphabetic language, Georgiou et al. (2008b) found a sizeable correlation between RAN (both colors and digits versions) and reading fluency in English, Greek, and Chinese 4th-graders, even if is worthy to note that some interesting correlations between RAN and reading accuracy also emerged for Greek and Chinese students only. The same pattern of results about reading fluency was also found in Finnish, English, and Chinese 4th-graders in a more recent work by Georgiou et al. (2016), and was further confirmed by an even more recent longitudinal comparison across orthographies by Landerl et al. (2019). Inclusive of five languages varying in orthographic consistency (English, French, German, Dutch, and Greek), this latter study tried to clarify in each language the contribution of RAN and PA to reading between the 1st and the 2nd grade, concluding that “*RAN taps a universal mechanism that is of similar relevance in learning to read across alphabetic orthographies, irrespective of differences in their complexity*” (Landerl et al., 2019, p. 230). Some opposite findings emerged for PA, whose relation to reading was not confirmed in all the orthographies considered (Landerl et al., 2019). This can be due to the different consistency of orthographies and their phonological complexity, and this is the reason why we do not consider PA as a valid reading predictor for the present study.

The recent meta-analytic work of Araújo and Faísca (2019), in line with Carioti et al. (2021), supports these results showing a systematic deficit of DD readers in the RAN task across orthographies stable with age.

The universal reliability of RAN as a marker of DD suggests that RAN can also be considered a candidate clinical marker of reading disorders in MLC, that is, in those children in which reading problems cannot be interpreted as a risk index, due to language disadvantage.

### The Present Study

As explained in previous sections, the gap of knowledge concerning reading outcomes of MLC, together with the lack of *ad hoc* created assessment instruments of reading skills in this specific population, make it difficult to discern between a neurodevelopmental reading disorder and reading problems due to a lack of language proficiency. Indeed, considering the role of language and the intrinsic linguistic nature of the reading process, one might ask how it could be possible to detect early signals of a reading disorder, regardless of the minority language spoken by students and the different cumulative exposure to the minority and majority language. A promising way is to find a reading-independent marker of DD, capable of identifying the neurodevelopmental disorder and distinguishing it between other linguistic and contextual issues, in the same vein as Vender et al. (2016) and Guasti et al. (2020) for children with developmental language disorders.

In the current study, we test whether the RAN task might be an effective tool to reach this goal. Thus, this study aims to explore whether MLC and monolingual Italian children without neurodevelopmental reading deficits reach similar performances in rapid naming, despite the MLC’s lower reading performances.

For these reasons, we included in our study (1) a group of monolingual poor readers (mGR), (2) a group of monolingual poor readers (mPR), and (3) a group of MLC.

According to the pieces of evidence discussed so far, the study will try to answer the following research questions:

• Does MLC struggle in reading as compared to mGR? Is their pattern of performance similar to the one observed in mPR?
• Is our new version of RAN (RAN-Shapes) capable of distinguishing mGR from mPR?
• Does mPR show a lower performance when compared with mGR at the RAN task? Does MLC differ from mPR, though showing a level of performance similar to mGR?

The last one is the crucial point of our investigation: based on the hypothesis that reading difficulties of MLC are related to a linguistic vulnerability and not to a learning disorder, we expect that (i) performances of mGR and MLC will be comparable, (ii) while only the mPR group will obtain poorer performance.

An *ad hoc* RAN-Shapes task has been developed to test our hypotheses. This new format of the RAN task, which requires the naming of five standard shapes (heart–circle–triangle–square–star), has been created with some novel and original features to avoid issues related to linguistic proficiency and to ensure an easy and enjoyable computerized administration for children.

## Materials and Methods

### Participants

One hundred and twenty-seven students from the primary school participated in the study. Of those, 46 were 3rd graders (24 F; mean age = 8.65, SD = 0.35), 41 were 4th graders (19 F; mean age = 9.52, SD = 0.32), and 40 were 5th graders (28 F; mean age = 10.66, SD = 0.3). The participants were recruited in the “I.C. Della Torre” primary school of Chiavari (Genova) and the Center of Developmental Neuropsychology – ASUR Marche, Pesaro.

Based on their familial linguistic context and their reading performances, students were divided into three groups: a group of native Italian speakers with typical development (*N* = 64), defined as mGR, a group of bilingual students born in Italy from foreign parents (*N* = 43), defined as MLC, and a group of monolinguals with reading scores below 1.5 standard deviations (for reading speed), and/or below the 10th percentile (for reading accuracy) in at least two reading measures (*N* = 20). This group was classified as mPR.

Groups were matched for age and IQ: the non-verbal reasoning score, measured by Raven’s matrices (Raven, 1956), was higher than the 85th percentile for all students (shown in Table 1 for participants’ demographic information).

**TABLE 1:** Participants’ demographic information.

Class	Group	*N*	Male/Female	Age [years (±SD)]	Age [months (±SD)]	Non-verbal reasoning [average raw score (±SD)]
*3rd grade*	mGR	17	8/9	8.57 (±0.33)	102.82 (±3.96)	33.41 (±1.42)
	mPR	8	3/5	8.99 (±0.25)	107.88 (±3)	31 (±2.31)
	MLC	21	11/10	8.59 (±0.35)	103.1 (±4.15)	33 (±2.02)
*4th grade*	mGR	24	13/11	9.47 (±0.32)	113.62 (±3.79)	33.83 (±1.27)
	mPR	7	4/3	9.79 (±0.14)	117.43 (±1.72)	33.29 (±1.80)
	MLC	10	5/5	9.47 (±0.33)	113.6 (±4.01)	33.3 (±2.06)
*5th grade*	mGR	23	7/16	10.61 (±0.31)	127.3 (±3.66)	34.78 (±1.41)
	mPR	5	2/3	10.57 (±0.28)	126.8 (±3.35)	33.80 (±2.17)
	MLC	12	3/9	10.78 (±0.27)	129.42 (±3.2)	34.67 (±1.78)

The students belonging to the MLC group were all born in Italy. They had at least one foreign parent (information about the different minority languages is reported in Supplementary Table 1). Almost everyone reported daily listening to a second language at home while using Italian mainly in the school context. Therefore, all of them could be considered as simultaneous bilinguals (Bialystok, 2001).

Twelve out of 20 students in the RD group were diagnosed as having a DD by professional neuropsychologists of the Center of Developmental Neuropsychology, three had a DD certification diagnosis elsewhere, and five were reported as having reading difficulties by teachers.

None of these children were identified as having psychiatric, emotional, or sensory disabilities, and all participants had normal or corrected-to-normal visual acuity. According to the World Medical Association Declaration of Helsinki’s ethical principles, the signed informed consent was obtained from parents, and children gave their verbal consent to the participation.

The study has been approved by the Ethical Committee of the University of Urbino Carlo Bo (prot. Num. 11, 20 August 2018).

### Cognitive Assessment

A short battery of cognitive and reading measures was administered to all participants during the first testing session. In particular, the reading tasks included are widely used in Italy for the assessment of learning disorders:

• *Raven’s Matrices*: Colored Progressive Matrices (CPM; Raven, 1956; Belacchi et al., 2008) were used to assess non-verbal reasoning. The test is standardized for children between 6 and 10 years and comprises three scales of 12 items, for a total of 36 matrices, and reported a good level of reliability (α = 0.82), as reported by Carlson and Jensen (1981).
• *Single word and pseudoword reading tasks* were assessed using the Battery for the assessment of Developmental Dyslexia and Dysorthographia-2 (DDE-2 Battery; Sartori et al., 2007). For each task, the total reading seconds and the number of errors were assessed for each student for the clinical evaluation, while accuracy (in percentage) and reading speed (in syllable/seconds) were used in the analyses. The test–retest reliability for these reading tasks satisfies the psychometric standards. Sartori et al. (2007) reported an *r* = 0.77 for speed and *r* = 0.56 for accuracy, while the concurrent validity varies from *r* = 0.74 to *r* = 0.96. The discriminant validity comparisons that meet 83% are also considered good for psychometric standards. The battery is the one most used in Italy for assessing reading and is required as preferential for the diagnosis of DD by the Consensus Conference Guidelines AID (2007).
• *Text reading* was assessed using the short stories in the battery MT-3 and MT-3 Clinica (Cornoldi and Colpo, 1981; Cornoldi et al., 1998; Cornoldi and Carretti, 2016). In this case, the parameters of time and accuracy were considered separately both for clinical evaluation and data analysis, as reported for word and pseudoword reading. The mean test–retest reliability reported for texts of this battery for all grades is 0.85 (α = 0.60; Cornoldi and Colpo, 1981). As the DDE-2 battery, the MT test is also included in tests required for the diagnosis of DD by the Consensus Conference Guidelines AID (2007).

### Experimental Task and Procedure

In our RAN-Shapes task, we asked participants to name as rapidly as possible five standard shapes (heart–circle–triangle–square–star) repeated in grids of different rows and columns. The choice to use shapes was due to two different reasons: (i) the need to limit the lexical access requested by the task to few stimuli easy to back up in memory and recall, for avoiding the influence of different language proficiency levels; (ii) the need to avoid possible effects of different levels of naming automatization in bilinguals and Italian monolingual children. Note that Åvall et al. (2019) observed higher automatization for alphanumeric tokens in school-age due to their salience in everyday school life. Since this effect could be affected by the access of word forms in different languages, as in bilingual readers, we preferred testing participants by using a set of generally less automatized stimuli.

As mentioned above, shapes were included into three different matrices of the same dimension, but that could vary for the number of shapes inscribed in or perceptual properties (i.e., the texture in background).

**FIGURE 1:**
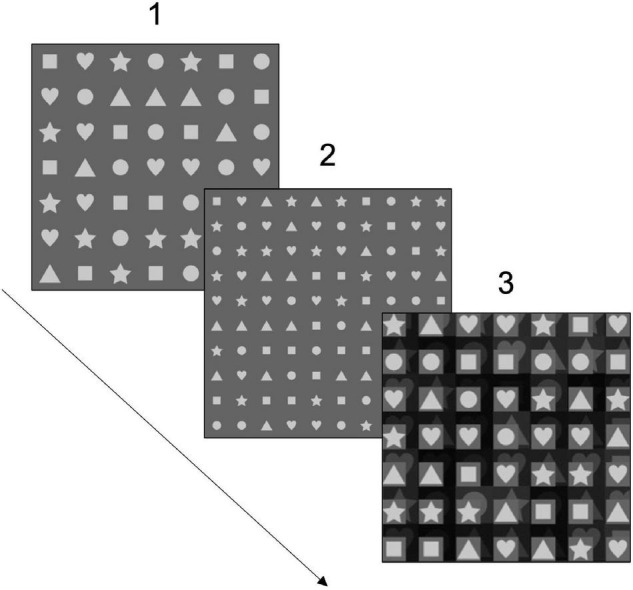
A representation of the Rapid Automatized Naming (RAN)-Shapes task. A first 7*7 matrix (1) to name is delivered to participants, followed by a second 10*10 matrix (2), which is more difficult because of the smaller shapes to identify, and by a third 7*7 matrix (3), which is characterized by background visual interference. For each matrix, 30 s are given to participants to name as many shapes as possible correctly.

Our RAN-Shapes task consists of three trials:

- *the RAN matrix 1* is a 7*7 grid with a total of 49 shapes;
- *the RAN matrix 2* is a 10*10 grid of the same size as the first, in terms of pixels, but with a total of 100 shapes inscribed in, which are, thus, smaller and closer to each other, requiring a higher grade of cognitive demand and to elicit the crowding effect;
- *the RAN matrix 3* is a 7*7 grid equal to the first one, for its size and number of stimuli (also 49 in total), but with a background visual interference, conceived to test the sensitivity to crowding effect and/or perceptual interference (shown in Figure 1).

As can be noticed, the three matrices involved two different sources of difficulties; in particular, they aimed to test whether a higher demand, represented by a higher density of stimuli to name or attentional burden (i.e., visual crowding), could affect naming speed. Additionally, they allowed clarifying whether task-automatization could occur while performing the three matrices in a fixed order (namely, RAN matrixes 1, 2, and 3).

In contrast with the classic version of Denckla and Rudel (1976) in our RAN, some of the shapes are repeated to test whether the need to articulate a similar or, in this case, the same phonological output would interfere with the naming speed (as suggested by Jones et al., 2013). Moreover, we decided to record the naming speed in terms of accurately named shapes in 30 s (×3 matrices)^3^ to provide a quick and soft administration, with the advantage of also avoiding a strain effect in participants. As a measure of fluency in reading, the effectiveness of this timed measure turned out to be equal to the untimed one, as pointed out in the meta-analysis by Carioti et al. (2021).

The experiment took place as follows: children were individually evaluated at school or the clinical center, in a quiet room, in two separate sessions: (i) the first session of cognitive assessment, and (ii) a second session in which the experimental RAN-Shapes task was administered to children. The RAN-Shapes task has been developed and administered in the Matlab 2018b environment using a PC DELL Inspiron 15 5000, with a 15.6 inches screen, Intel Core*™* i7-1165G7 driver, and Windows Home 10 Operative System. During the RAN-Shapes task, each participant was set in front of the PC and asked to wear headphones (Philips Bass + SHL3075WT/00 with integrated microphone).

### Data Analysis

Data analyses were performed in the R environment (R Core Team, 2019).

We first checked whether the Socio-Economic Status (SES) has an influence on reading and naming skills using the Intraclass Correlation Coefficient (ICC) in the R package ICC (Wolak, 2015). The SES was computed according to parents’ occupation. The occupations were classified using the nomenclature of International Standard Classification of Occupations (ISCO team, International Labour Office), and coded along 10 areas.

Based on this classification, the occupation of mothers and fathers were collapsed in a unique score, resulting in a three-way classification (high–medium–low level of SES; shown in Supplementary Table 2).

After this preliminary passage, to explicitly test our first working hypothesis, between-group performances on each reading measure were compared using Generalized Linear Models (GLMs): as Italian-monolingual participants were assigned to mGR or mPR group based on reading performances, the principal aim of this first analysis was to test whether MLC reported lower reading skills, compared to mGR, and whether their difficulties were closer to those of mPR and, if so, more impaired in word and text reading or pseudoword reading. Accordingly, 3*3 GLMs were implemented using the *glm* routine of the “stats” package, with the variable “group” (mGR – MLC – mPR) and “class” (3rd – 4th – 5th grades) as predictors. When reading measures (indices of accuracy and fluency in word, pseudoword, and text reading) were not normally distributed, some transformations were applied: data distribution was transposed and moved to the positive axis by adding two scores to test the fitting with a gamma distribution. When this was not effective, a log_10_ transformation has been applied. In case of a significant main effect, together with a not significant Levene Test, *post-hoc* comparisons corrected for multiple comparisons with Tukey’s method were implemented. When significant, the interaction effects among groups and classes were explored using the *TestInteractions* routine of the “phia” package (De Rosario-Martinez, 2013); *post-hoc* analysis of interaction effects that are eventually found are reported as corrected, for false discovery rate correction (*fdr*; Benjamini and Hochberg, 1995; Genovese and Wasserman, 2002; Thissen et al., 2002).

To better explore the RAN-reading relationship, in the second step, the correlational patterns between reading measures, performances at the three RAN’s matrices, and the severity of the reading deficit were investigated on the monolingual sample (mGR + mPR), by using the *cor.test* function of the “stats” package.

The severity of the reading deficit was defined as the number of reading parameters in which a participant reported a performance under 1.5 SD or the 10th percentile. We tested reading skills through three tasks (word, pseudoword, and text reading), obtaining a fluency and an accuracy index for each one; the severity level ranging from 0 to 6 was, thus, assigned for each impaired reading index.

For testing our third research hypothesis, logistic regressions were then implemented to explore if the performance at the RAN task can predict whether a participant belongs either to the mGR or to the mPR. Accordingly, the logistic regression was designed with the variable “Group” as dependent and each matrix of RAN as a predictor.

As the final step, to test our third working hypothesis, we compared the performances of the three groups in the three matrices of RAN through a General Linear Mixed Model (GLMM), using the *lmer* routine of the “lme4” package (Bates et al., 2014). In particular, the model was designed using the identification code of participants as intercepts and matrix type (RAN_matrix1_ – RAN_matrix2_ – RAN_matrix3_), group (mGR – MLC – mPR), and class (3rd – 4th – 5th grade) as fixed effects.

## Results

The ICC values computed on both the reading and RAN measures suggested that the SES is not a clustering factor for our sample on the variables of interest (shown in Table 2).

**TABLE 2:** Intraclass Correlation Coefficient (ICC) calculated to evaluate whether the three levels of Socio-Economic Status (SES) (high–medium–low) clustered reading and Rapid Automatized Naming (RAN) performances.

	Tasks	ICC	Lower CI	Upper CI	*N*	*k*	var w	var a
*Reading fluency (syll./sec.)*	*Word reading*	−0.006	−0.03	0.29	3	25.39	0.88	−0.005
	*Pseudoword reading*	0.01	−0.02	0.42	3	25.39	0.37	0.005
	*Text reading*	−0.02	−0.03	0.18	3	25.39	1.2	−0.02
*Reading accuracy (% of accuracy)*	*Word reading*	−0.009	−0.03	0.27	3	25.39	15.58	−0.14
	*Pseudoword reading*	−0.02	−0.03	0.11	3	25.39	100.6	−2.74
	*Text reading*	−0.01	−0.03	0.2	3	25.39	7.41	−0.13
*RAN-Shapes*	*Matrix 1*	−0.02	−0.03	0.12	3	25.39	53.06	−1.37
	*Matrix 2*	−0.02	−0.03	0.11	3	25.39	52.44	−1.42
	*Matrix 3*	−0.03	−0.03	0.02	3	25.39	60.93	−2.09

### Reading Proficiency of Monolingual Poor Readers and Minority-Language Children

**FIGURE 2:**
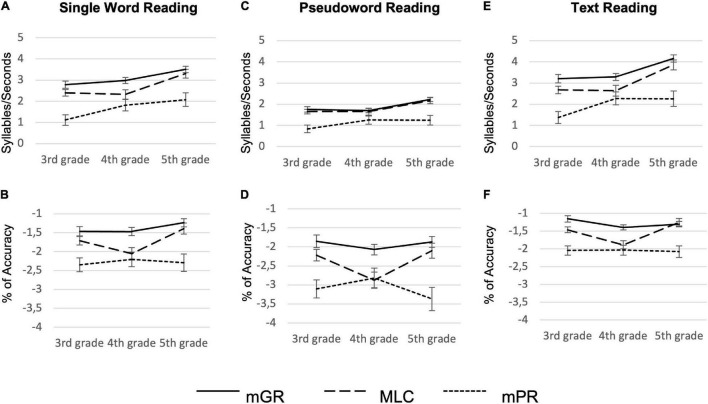
Reading performances of monolingual Good Readers (mGR), minority-language children (MLC), and monolinguals Poor Readers (mPR). Panels **(A,C,E)** display reading accuracy (percentage of accuracy), while panels **(B,D,F)** display reading fluency (syllables/seconds).

Between-group comparison in reading performances revealed a significant main effect of group and class effect in each reading measure: the mPR group significantly underperformed compared both to mGR and MLC in all reading tasks (Figure 2 graphically shows the descriptive pattern of results, Tables 3, 4 report the summary of the statistical analyses, and Table 5 shows descriptive statistics).

**TABLE 3:** Simple and interaction effects emerged by the Generalized Linear Models (GLMs) run on reading measures.

	Reading measure	Effect	*X* ^2^	df	*p*-Value	η^2^	Adjusted *R*^2^
*Reading fluency (syll./sec.)*	*Word reading*	Group	60.43	2	<0.001***	0.31	
		Class	29.46	2	<0.001***	0.13	
		Group*Class	3.74	4	0.44	0.01	0.43***
	*Pseudoword reading*	Group	36.77	2	<0.001***	0.22	
		Class	23.66	2	<0.001***	0.12	
		Group*Class	3.66	4	0.45	0.02	0.32***
	*Text reading*	Group	55.59	2	<0.001***	0.29	
		Class	37.86	2	<0.001***	0.16	
		Group*Class	5.47	4	0.24	0.22	0.44***
*Reading accuracy (% of accuracy)*	*Word reading^+^*	Group	45.03	2	<0.001***	0.27	
		Class	7.48	2	0.02*	0.04	
		Group*Class	4.98	4	0.28	0.02	0.29***
	*Pseudoword reading^+^*	Group	43.55	2	<0.001***	0.25	
		Class	3.55	2	0.16	0.21	
		Group*Class	8.14	4	0.08	0.04	0.27***
	Text reading^+^	Group	64.34	2	<0.001***	0.31	
		Class	9.81	2	0.007**	0.04	
		Group*Class	10.96	4	0.02*	0.05	0.37***

^+^gamma distribution, ***p < 0.001, **p < 0.01, *p < 0.05.

**TABLE 4:** Results of the Leven’s Test and *post hoc* statistic comparisons run for exploring significant effects emerged from the GLMs run on reading measures.

Levene’s Test				*Post hoc* comparisons							
Fluency (syll./sec.)	*F*	df	*p*-Value	Main effect considered	Correction		Contrast	*z* ratio		*p*-Value	Cohen’s *d*
*Word reading*	0.87	2-124	0.41	Group	Tukey		mGR-mPR	−7.67		<0.001***	2.12
							mGR-MLC	2.83		0.012*	–0.6
							mPR-MLC	−5.1		<0.001***	1.28
*Pseudoword reading*	1.99	2-124	0.14	Group	Tukey		mGR-mPR	−5.97		<0.001***	1.62
							mGR-MLC	0.66		0.78	–0.19
							mPR-MLC	−5.1		<0.001***	1.34
*Text reading*	2.31	2-124	0.1	Group	Tukey		mGR-mPR	−7.5		<0.001***	2.02
							mGR-MLC	2.98		0.007**	–0.61
							mPR-MLC	−4.83		<0.001***	1.14

**Accuracy (%)**	***F***	**df**	***p*-Value**	**Main effect considered**	**Correction**		**Contrast**	***z* ratio**		***p*-Value**	**Cohen’s *d***

*Word reading*	4.22	2-124	0.016*	Group	Tukey		mGR-mPR	6.67		<0.001***	–1.93
							mGR-MLC	−3.16		0.004**	0.59
							mPR-MLC	3.92		<0.001***	–1.06
*Pseudoword reading*	2.98	2-124	0.054	Group	Tukey		mGR-mPR	6.58		<0.001***	–1.76
							mGR-MLC	−3.36		0.002**	0.56
							mPR-MLC	3.69		<0.001***	–1.07
				
				**Significant effect**	**Correction**	**Class**	**Contrast**	***X* ^2^**	**df**	***p*-Value**	**Difference**
				
*Text reading*				Group*Class	fdr	3rd grade	mGR-mPR	31.61	1-118	<0.001***	–4.69
					fdr		mGR-MLC	6.33	1-118	0.012*	–1.29
					fdr		mPR-MLC	14.28	1-118	0.001***	3.39
					fdr	4th grade	mGR-mPR	16.44	1-118	<0.001***	–4.01
					fdr		mGR-MLC	12.56	1-118	<0.001***	–3.39
					fdr		mPR-MLC	0.45	1-118	0.45	0.62
					fdr	5th grade	mGR-mPR	18.26	1-118	<0.001***	–4.31
					fdr		mGR-MLC	0.15	1-118	0.69	0.21
					fdr		mPR-MLC	17.83	1-118	<0.001***	4.52

***p < 0.001, **p < 0.01, *p < 0.05.

**TABLE 5:** Descriptive statistics of reading performances of students in each group and grade.

		*mPR*			*mGR*			*MLC*		
Task	Grade	*N*	Mean	SD	*N*	Mean	SD	*N*	Mean	SD
Word reading (syll./sec.)	3	8	1.11	0.41	17	2.78	0.72	21	2.40	0.73
Word reading (% acc.)	3	8	90.63	4.13	17	96.90	3.37	21	95.32	4.53
Pseudoword reading (syll./sec.)	3	8	0.83	0.32	17	1.76	0.60	21	1.65	0.58
Pseudoword reading (% acc.)	3	8	76.82	10.94	17	94.00	5.93	21	89.19	10.80
Text reading (syll./sec.)	3	8	1.38	0.39	17	3.21	0.74	21	2.67	0.83
Text reading (% acc.)	3	8	93.94	2.56	17	98.63	1.24	21	97.34	1.94
RAN_matrix1_	3	8	24.38	8.09	17	30.53	5.04	21	33.24	6.92
RAN_matrix2_	3	8	23.50	6.35	17	31.53	5.65	21	32.62	6.86
RAN_matrix3_	3	8	26.25	6.61	17	34.06	5.86	21	38.10	7.99
Word reading (syll./sec.)	4	7	1.81	0.62	24	2.98	0.54	10	2.32	0.60
Word reading (% acc.)	4	7	92.60	2.71	24	97.21	1.98	10	93.04	4.76
Pseudoword reading (syll./sec.)	4	7	1.25	0.38	24	1.70	0.31	10	1.65	0.38
Pseudoword reading (% acc.)	4	7	83.04	10.99	24	92.36	5.20	10	82.50	9.63
Text reading (syll./sec.)	4	7	2.27	0.75	24	3.29	0.65	10	2.64	0.66
Text reading (% acc.)	4	7	93.78	3.71	24	97.80	1.33	10	94.40	4.56
RAN_matrix1_	4	7	27.57	6.85	24	33.50	6.35	10	33.70	3.89
RAN_matrix2_	4	7	26.43	4.43	24	32.71	6.31	10	31.00	3.33
RAN_matrix3_	4	7	34.14	4.22	24	38.21	7.40	10	37.50	5.25
Word reading (syll./sec.)	5	5	2.08	0.51	23	3.50	0.80	12	3.31	1.03
Word reading (% acc.)	5	5	91.43	3.55	23	98.25	1.60	12	97.32	3.00
Pseudoword reading (syll./sec.)	5	5	1.24	0.29	23	2.22	0.57	12	2.17	0.66
Pseudoword reading (% acc.)	5	5	70.83	11.88	23	94.11	4.64	12	92.36	7.34
Text reading (syll./sec.)	5	5	2.26	0.50	23	4.16	0.90	12	3.86	1.29
Text reading (% acc.)	5	5	93.77	2.23	23	98.08	1.47	12	98.29	1.41
RAN_matrix1_	5	5	29.80	4.32	23	39.65	5.69	12	37.08	6.56
RAN_matrix2_	5	5	28.20	2.86	23	38.74	6.68	12	37.33	6.17
RAN_matrix3_	5	5	33.20	4.76	23	43.91	6.04	12	41.75	5.40

Concerning the reading fluency (in terms of syllables/seconds), when Tukey-corrected *post hoc* comparisons were explored, a significant difference between mGR and mPR in single word reading (*z* = 2.83, *p* = 0.012, *d* = −0.6) and text reading (*z* = 2.98, *p* = 0.007, *d* = −0.61) emerged; however, we did not find any difference in pseudoword reading (*z* = 0.66, *p* = 0.78, *d* = −0.19).

A slightly different pattern of results emerged for reading accuracy, although, in this case, MLC also made a higher rate of errors in pseudoword reading (Tables 3, 4). In particular, we found a significant difference between MLC and mGR in word (*z* = −3.16, *p* = 0.004, *d* = 0.59) and in pseudoword reading accuracy (*z* = −3.36, *p* = 0.002, *d* = 0.56). Interestingly, no main effect of class was found for pseudoword reading accuracy [*X*^2^_(2)_ = 3.55, *p* = 0.16, *h*^2^ = 0.21]. Moreover, concerning text reading accuracy, a class-by-group interaction effect emerged [*X*^2^_(4)_ = 10.96, *p* = 0.02, *h*^2^ = 0.05]; in particular, exploring *fdr* corrected *post-hoc*, a significant difference between mGR and MLC emerged only in 3rd [*X*^2^_(1)_ = 6.33, *p* = 0.012] and 4th grade [*X*^2^_(1)_ = 12.56, *p* < 0.001], but not in 5th grade [*X*^2^_(1)_ = 0.15, *p* = 0.695].

### Rapid Automatized Naming-Reading Relationship

The correlational matrix between reading skills, the performance at the three RAN-Shapes matrices, and the number of reading deficits reported (max. 6) is reported in Table 6. As it can be seen, moderate correlations emerged between all reading measures and the three RAN matrices, and, in general, the number of deficits reported in reading measures correlates with RAN (RAN_matrix1_: rho = −0.4, *p* < 0.001; RAN_matrix2_: rho = −0.49, *p* < 0.001; RAN_matrix3_: rho = −0.38, *p* < 0.001).

**TABLE 6:** Matrix of non-parametric correlation between matrices of the RAN-Shapes and reading measures, and between matrices of the RAN-Shapes and the degree of severity of the reading deficit reported by monolingual Italian readers [monolingual Good Readers + monolingual Poor Readers; (mGR + mPR)].

		RAN_matrix1_		RAN_matrix2_		RAN_matrix3_	
		*Rho*	*p-Value*	*Rho*	*p-Value*	*Rho*	*p-Value*
*Reading fluency (syll./sec.)*	Word reading	0.58	<0.001***	0.6	<0.001***	0.57	<0.001***
	Pseudoword reading	0.47	<0.001***	0.53	<0.001***	0.52	<0.001***
	Text reading	0.52	<0.001***	0.61	<0.001***	0.58	<0.001***
*Reading accuracy (% of accuracy)*	Word reading	0.39	<0.001***	0.45	<0.001***	0.41	<0.001***
	Pseudoword reading	0.26	0.001***	0.35	<0.001***	0.32	<0.001***
	Text reading	0.3	<0.001***	0.36	<0.001***	0.28	<0.001***
*Severity of the reading deficit*	Severity of the reading deficit	−0.4	<0.001***	−0.49	<0.001***	−0.38	<0.001***

***p < 0.001, **p < 0.01, *p < 0.05.

Notably, as depicted in Table 6, stronger correlations were found between RAN and reading fluency measures, while the negative correlations with the severity of reading deficit (i.e., the number of deficits for each participant) suggest that the more severe the deficit, the lower the number of correctly named items in 30 s.

### Rapid Automatized Naming as a Predictor of Reading Proficiency

Logistic regressions were then implemented to test whether each RAN matrix was able to predict the group assignment of Italian native participants in terms of mGR or mPR.

All RAN matrices resulted as good predictors of reading difficulties (RAN_matrix1_: *z* = −3.67, *p* < 0.001, OR = 1.18; RAN_matrix2_: *z* = 3.93, *p* < 0.001, OR = 1.26; RAN_matrix3_: *z* = −3.64, *p* < 0.001, OR = 1.15).

### Rapid Automatized Naming as a Marker of Developmental Dyslexia

Results of the GLMM used to compare performances of groups at the three matrices of RAN in each class showed: a significant main effect of the matrix type [*X*^2^_(2)_ = 132.03, *p* < 0.001], a significant main effect of class [*X*^2^_(2)_ = 34.36, *p* < 0.001] and a significant main effect of group [*X*^2^_(2)_ = 32.06, *p* < 0.001].

**FIGURE 3:**
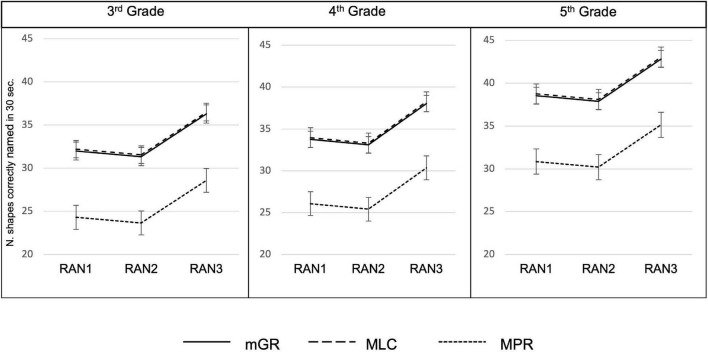
Performances of mGR, MLC, and mPR at the three matrices of the RAN-Shapes in 3rd, 4th, and 5th grade.

In particular, from *post-hoc* comparisons adjusted for Tukey’s correction, significant differences between the second and the third matrices emerged in all groups [matrix2–matrix3: *t*_(244)_ = −10.57, *p* < 0.001] and the 5th graders reported a better performance than both 3rd graders [*t*_(118)_ = −5.49, *p* < 0.001] and 4th graders [*t*_(118)_ = −3.96, *p* < 0.001]. Moreover, the mPR and mGR groups were significantly different in all matrices of RAN [*t*_(122)_ = −5.48, *p* < 0.001], and the same can be said for the comparison between mPR and MLC [*t*_(118)_ = −5.35, *p* < 0.001], but no differences between mGR and MLC emerged [*t*_(118)_ = −0.19, *p* = 0.97]. The results are depicted in Figure 3 (see Table 5 for descriptive statistics stratified for groups and grades).

## Discussion

In the current study, we tested the RAN task, i.e., one of the universal predictors of reading difficulties, on three groups of participants: mGR, mPR, and MLC. In line with the literature, the results revealed that mGR outperformed mPR in the RAN task. Nevertheless, most importantly, we observed that MLC did not differ from mGR, though displayed a lower reading performance. In general, such findings suggest that the RAN task is a reliable marker for identifying the risk of learning disorders in monolinguals. Additionally, they indicate that it could also be adopted in the bilingual population without introducing any systematic disadvantage associated with language use and exposure. Taken together, the results of the reading and the RAN-Shapes tasks suggest that, in MLCs, a low reading performance might be ascribed to a language disadvantage rather than to a reading disability. We will start by discussing the results of the MLC group.

The MLC participants underperformed mGRs in all the reading accuracy measures (shown in Figures 2B,D,F) while showing no difference in pseudoword reading fluency (shown in Figure 2C). This pattern is consistent with previous results based on an Italian study by Bellocchi et al. (2016). In this study, in the 2nd graders, bilingual children underperformed their monolingual peers in word, pseudoword, and text reading accuracy, while like in our study, the group of bilingual children did not differ from monolinguals on pseudowords reading speed. This result seems particularly relevant if one considers that the deficit in pseudoword reading fluency is one of the most common manifestations of DD in both children and adults across European orthographies (refer to Ziegler et al., 2010; Landerl et al., 2013; Araújo et al., 2015; Araújo and Faísca, 2019; Parrila et al., 2020; Carioti et al., 2021). The fact that MLC participants did not show a fluency deficit is particularly relevant as it may support our hypothesis: the MLC’s reading difficulties are not necessarily due to a neurodevelopmental condition, rather, they might represent the behavioral manifestation of a language disadvantage (Bialystok et al., 2008).

Indeed, it is well-established that a between-group difference, on average, does not necessarily imply a group deficit from a neuropsychological point of view. The MLC’s performance may be lower than the average score for mGR, but still within the normal range (Capitani and Laiacona, 1988; Capitani, 1997; Gallucci et al., 2017). This should be kept in mind when looking at the between-group comparisons reported here.

However, if one focuses on the MLCs’ performance in pseudoword reading accuracy (Figure 2D) in the 4th grade, a clear overlap between MLC and mPR emerged (see Table 5 for descriptive statistics). This could suggest that some participants, particularly in 4th grade (shown in Figure 2D), struggle in the grapheme-to-phoneme conversion due to a neurodevelopmental condition, consequently lowering the average performance of the entire group. This scenario should be better explored at the single-subject level with *ad hoc* created tests.

Another relevant result comes from SES. As observed, a low, medium, or high-level SES did not impact on reading proficiency and, thus, considering the peculiar reading pattern observed in these children, we could assume that reading difficulties reported by MLC are not related to aspects such as parents’ occupation, rather, to the use and experience of more than one language in the everyday life.

As already suggested, a hypothesis could be that the reading difficulties observed in the MLC are ascribable to limited access to the lexical information in the majority language. Different perspectives can explain this lexical weakness: one cause could be simply related to a reduced vocabulary in the L2 (as already suggested by Bellocchi et al., 2016), while another hypothesis suggests that simultaneous engagement of both languages would interfere with the linguistic production and, thus, with correct and fast recognition of the word (Dijkstra, 2005; Titone et al., 2011). The latter case would also support the idea of an integrated mental lexicon in which lexical representations of all the languages known are stored together, where a non-selective activation during language processing would cause a cross-linguistic interference for specific words (Dijkstra and Van Heuven, 2002; Schwartz et al., 2008). Unfortunately, we observed only weakness in lexical recognition for Italian words, therefore, we cannot provide support for this claim; further studies are needed to better explore this issue about reading.

Anyway, in this regard, it is important to note that we observed a higher proficiency in text reading accuracy by bilingual students of higher grade (shown in Figure 2D): this may support the importance attributed to L2 exposure in influencing bilinguals’ linguistic and literacy skills (Monaghan et al., 2017). This would be further supported by the fact that our MLC were (almost) all simultaneous bilinguals. Therefore 5th-graders that were exposed to L2 (Italian) for a longer time, showed better skills in integrating semantic information that allowed to enhance reading efficacy. We are aware that this observation must be further tested using longitudinal designs to check whether sentence/text reading shows improvement as a function of age, exposure to the majority language, and consequent language expertise.

To sum up, our pattern of results supports the idea that minority-language primary students’ literacy skills might be poorer than those of monolinguals. However, even though from a behavioral point of view their reading performance might seem similar to those of dyslexic readers, if one considers the reading profile (in particular for what concerns pseudo-word reading fluency), together with the performance at the RAN-Shape task, some interesting differences emerged. These differences represent some of the clinical markers to be considered when assessing a bilingual primary student.

### The Rapid Automatized Naming-Shapes as a Marker of Reading Difficulties in Monolinguals and Minority-Language Children

The logistic regressions revealed that all the three matrices of our RAN-Shapes allowed us to predict whether participants belonged to the group of monolingual good (mGR) or poor readers (mPR). Interestingly the second matrix, i.e., the one with a reduced inter-letter spacing, reported the higher odd ratio and resulted, thus, as the strongest predictor of reading difficulties. This result is in line with previous studies about the crowding effect and its role in slowing down the performance of mPR readers (Spinelli et al., 2002; Yu et al., 2007; Martelli et al., 2009). However, it is worth noting that this visuo-attentional effect only occurs when the inter-letter spacing (Martelli et al., 2009) was reduced and not in the case of background interference (i.e., in the third matrix).

Taken together, all these findings showed that our version of RAN, the RAN-Shapes, effectively discriminates between good and poor readers.

Moreover, the correlations between the performance at the RAN matrices and the variable “severity of the reading deficit,” ranging from a simple reading difficulty (<−1.5 SD in at least two reading parameters) to a severe deficit (<−1.5/−2 SD in all reading parameters), suggests that the lower the performance at the RAN-Shapes task, the more impaired the reading skills.

Although further studies are needed to better clarify whether the RAN-Shapes can isolate specific profiles of dyslexia (ex., cases sensitive to crowding effect), in general, we can suggest that the RAN-Shapes emerged as a reliable cognitive marker of reading difficulties and that the level of performance at this task might detect severe reading deficits.

Once empirically proved that our RAN-Shapes task is sensitive to reading difficulties, we tested it on bilingual participants. We assumed that if a reading neurodevelopmental disorder was at the basis of the low level of MLC performances in reading tasks, they would manifest a behavioral performance similar to the mPRs’ one, which is also in the three matrices of our RAN-Shapes task. This was not the case: the MLC did not show any difference at the RAN-Shapes task when compared with mGR, rather they differed from mPR. Indeed, their level of performance was higher than the one of mPR suggesting that the RAN-Shapes can be considered an unbiased marker of a neurodevelopmental reading disorder even in a multilingual subject. Additionally, we showed that the SES did not influence rapid naming, to further support the idea that the relationship between RAN and reading skills can be considered mainly cognitive, rather than influenced by familial and environmental variables (van Bergen et al., 2014).

This result has both clinical and educational implications: bilingual children may manifest difficulties in the acquisition of reading and a slowdown in automatization in the absence of a neurodevelopmental disorder, therefore, the adoption of the unbiased markers, such as our RAN-Shapes task, should be highly recommended in the clinical practice.

### Some Further Evidence: The Rapid Automatized Naming-Reading Relationship

We observed that MLC, though showing lower performances in lexical decoding and a higher rate of errors in pseudoword reading, were not affected in a naming task such as the RAN. This is somehow surprising if one considers both evidence about the lexical deficit in naming skills of bilinguals (Dijkstra, 2005; Titone et al., 2011; Bylund et al., 2019) and theories about the relationship between RAN and orthographic-lexical word retrieval (Bowers and Kennedy, 1993; Young and Bowers, 1995; Savage and Frederickson, 2005; Georgiou et al., 2010). Along these lines, the participants impaired in word recognition and text reading (a task in which orthographic analysis, lexical retrieval, and parafoveal information processing are needed as much as in the RAN task) should show an impairment in RAN too. In other words, if the RAN-reading relationship is due to orthographic processing and lexical retrieval (Bowers and Wolf, 1993; Bowers et al., 1994; Georgiou et al., 2010), children showing weakness in lexical aspects of reading, as our MLC readers, should be impaired in the RAN task too. Our results suggest that this is not the case.

Nevertheless, the theories that link RAN to phonological awareness and assume that the level of proficiency mediates performance in naming and in retrieving phonological code from long-term memory (Torgesen et al., 1997; Vellutino et al., 2004; Bowey et al., 2005; Clarke et al., 2005) do not seem to be fully supported by our results, as in our case, a higher degree of errors in phonological decoding reported by MLC compared to mGR was not associated with lower naming performances.

Whether the phonological or the orthographic accounts better explain the RAN-reading relationship has also been studied by assuming a cross-linguistic perspective; according to orthographic depth (Katz and Frost, 1992), if RAN is related to orthographic and lexical aspects of reading and language, then, the relationship between RAN and word recognition will be stronger in deep orthographies in which a lexical identification strategy is more suitable and, thus, better automated (Savage et al., 2018; refer to Devoto et al., 2021 for a review of neuroimaging evidence). The opposite assumption can be made about phonological decoding and its relationship with RAN in shallow orthographies (like Italian). Based on these predictions, the validity of the two perspectives (the phonological and the orthographic one) was investigated by observing performances at the RAN task in MLC and, specifically, the transfer between two languages at different depth levels that, unfortunately, returned conflicting results (Siddaiah et al., 2016; Yeung, 2016; Antzaka et al., 2018; Savage et al., 2018). Although some studies on bilinguals (ex. Yeung, 2016) seem to sustain the orthographic perspective while others do not exclude the influence of the phonological processing (e.g., Antzaka et al., 2018; Savage et al., 2018), the between-studies variability of language and the gap in orthographic depth between them make it hard to compare these works and, thus, to extract a unique conclusion. Our results, based on a shallow orthography such as Italian, showed that a lexical weakness was not associated with a RAN deficit; in contrast, the naming deficit was present only in mPRs who showed a deficit in the speed of grapheme-to-phoneme conversion. The MLCs, who were inaccurate in pseudoword reading, did not show such a pattern, suggesting that naming speed deficit cannot be exhaustively explained according to either the lexical or the phonological perspective only.

A third theory to explain the RAN-reading relationship identifies in automaticity and attention-based processes, the common aspects underlying reading and serial naming (Kail and Hall, 1994; Savage and Frederickson, 2005; Borokhovski, 2007). In this perspective, the automaticity can be conceived in two different perspectives: as (i) speed of processing (Kail and Hall, 1994; Borokhovski, 2007; Powell et al., 2007), i.e., the ability to quickly integrate the different processes involved in the task from lexical and phonological retrieval to the articulatory output, or as (ii) a progressive automatization of lexical access, namely, a progressive enhancement of speed, as a function of practicing (Georgiou and Stewart, 2013).

Current results based on bilinguals, together with the significant correlations found between the RAN and all measures of reading in monolingual readers, seem to support the first perspective concerning the speed of processing, especially, if we consider that the higher correlations emerged between the three matrices and reading fluency (Araújo et al., 2015; Araújo and Faísca, 2019).

Looking at the second interpretation, by observing the variation of mean pause time between the first and the second half of a RAN matrix, Georgiou and Stewart (2013) found no support for the idea of a progressive automatization of recall. Opposite to this view, we found a linear enhancement of naming fluency by considering our three matrices’ trajectories that were interpreted as proof of task automatization in all groups. This evidence seems to exclude the possibility that a deficit in progressively automatizing cognitive procedures underlies the reading impairment.

These results, opposite to those of Georgiou and Stewart (2013), can be explained by considering that we took into account another more general and straightforward measure of fluency (shapes named in 30 s) and that we observed a wider time window (three matrices). Based on our results, we cannot explain the naming deficit shown by mPR in terms of failure in automatizing the task’s execution. For this reason, considering the features of the groups involved, and the nature of the reading profile of each group, we suggest that our results are consistent with the hypothesis that a serial naming deficit is associated with a deficit in the fast integration of phonological, orthographic, lexical, and articulation processes causing a deficit in reading.

On the contrary, children without a learning disorder, though showing lexical difficulties in access and storage, do not usually exhibit an extensive dysfunction associated with a failure in the RAN task (Georgiou and Stewart, 2013).

## Conclusion

In the present study, we showed that a new version of the RAN task, the RAN-Shapes, is a reliable predictor of a reading deficit, allowing to target failures in rapidly integrating several underlying phonological- and orthographic-based processes that support reading. Our results indicate that the RAN-Shapes task can be used as a substantive criterion to disentangle between a reading deficit and a reading difficulty due to a linguistic weakness, as in the bilingual sample. Crucially, our RAN-Shapes task allowed us to discriminate between children with and without reading disorders, and in line with our results, it would, thus, also represent valid support in the cognitive assessment of bilingual students.

Therefore, it is important to consider current findings, when evaluating from a cognitive and neuropsychological point of view, the reading profile of bilingual readers in clinical services. Similarly, our results indicate the need for a programming educational path for the acquisition of literacy in bilinguals, including specific training of vocabulary and lexical skills. Future studies are necessary to better clarify what specific aspects of the reading deficit can be identified through its use.

Another intriguing further direction would be to test RAN-letters, digits, and our RAN-Shape task in the same sample of participants as some sources of evidence were put forward to support the idea that alphanumeric vs. non-alphanumeric stimuli may lead to different profiles of learning deficit (Donker et al., 2016).

As a final remark, we are aware that RAN is a single and specific cross-cultural cognitive marker of the reading deficit; however, according to the intrinsic multi-dimensional nature of developmental dyslexia (Pennington, 2006; Pennington et al., 2012; van Bergen et al., 2014; Surushkina et al., 2021), the research agenda should include the development of a more complex pool of cognitive reading-free tasks to also reliably assess reading-related skills in children speaking a minority language.

## Data Availability Statement

The raw data supporting the conclusions of this article are available on request to the corresponding author.

## Ethics Statement

The studies involving human participants were reviewed and approved by Ethical Committee for Human Experimentation (CESU), University of Urbino Carlo Bo. Written informed consent to participate in this study was provided by the participants’ legal guardian/next of kin.

## Author Contributions

DC, MB, NS, and MG contributed to the study’s conception and design. CT programmed the RAN-Shapes task in the Matlab environment. DC, MFM, MBR, and SC collected experimental data. DC, MD, RR, ST, and AM performed the cognitive assessment. DC performed statistical analyses and wrote the first version of the draft, while MB and MV revised it. All authors commented on previous versions of the manuscript and approved the final manuscript.

## Conflict of Interest

The authors declare that the research was conducted in the absence of any commercial or financial relationships that could be construed as a potential conflict of interest.

## Publisher’s Note

All claims expressed in this article are solely those of the authors and do not necessarily represent those of their affiliated organizations, or those of the publisher, the editors and the reviewers. Any product that may be evaluated in this article, or claim that may be made by its manufacturer, is not guaranteed or endorsed by the publisher.

## Footnotes

1
www.miur.gov.it

2 PISA is an international assessment administered by European countries every year to evaluate educational outcomes of students. The program is coordinated by the Organization for Economic Cooperation and Development (OECD).

3 Usually, the RAN score is recorded as the total amount of seconds to name all colors/digits/objects/letters.
