# Supplementary material for: Rapid Automatized Naming as a Universal Marker of Developmental Dyslexia in Italian Monolingual and Minority-Language Children
Source: Front Psychol. 2022 Apr 7;13:783775. doi: 10.3389/fpsyg.2022.783775 (PMC9021430; doi:10.3389/fpsyg.2022.783775)
Supplement: Supplementary file 1 [file Data_Sheet_1.PDF]

<u>Supplementary materials</u>
RAN as a universal marker of developmental dyslexia in Italian monolingual and minority language children

Desiré Carioti[1,2], Natale Stucchi[2], Carlo Toneatto[2], Marta Franca Masia[1], Martina Broccoli[1], Sara Carbonari[1], Simona Travellini[1, 3], Milena Del Monte[3], Roberta Riccioni[3], Antonella Marcelli[3], Mirta Vernice[1], Maria Teresa Guasti[2], Manuela Berlingeri[1,3,4]

Table S1

Parental country of origin and prevalence in minority language children (%)

**Father's Nationality**		**Mother's Nationality**	
Country	*%*	*Country*	*%*
Albania	32.65	Albania	34.69
Ecuador	16.33	Ecuador	16.33
China	8.16	China	8.16
Italy	8.16	Peru	8.16
Not Reported	6.12	Ukraine	8.16
Peru	6.12	Marocco	6.12
Marocco	4.08	Moldavia	6.12
Moldavia	4.08	Romanian	4.08
Ukraine	4.08	Italy	2.04
Azerbaijan	2.04	Saint-Domingue	2.04
Portugal	2.04	Columbia	2.04
Romanian	2.04	Spain	2.04
Saint-Domingue	2.04		
Spain	2.04		
Tot.	100 %		100 %

Table S2

International Standard Classification of Occupations and levels of SES extracted on its bases

**Occupation**	**Classification**	**SES-Level**
Managers	1	High
Professionals	2	High
Technicians and Associate Professionals	3	Medium
Clerical Support Workers	4	Medium
Services and Sales Workers	5	Medium
Armed Forces Occupations	0	Medium
Skilled Agricultural, Forestry and Fishery Workers	6	Low
Craft and Related Trades Workers	7	Low
Plant and Machine Operators and Assemblers	8	Low
Elementary Occupations	9	Low
